# Relationship of patient safety culture with factors influencing working environment such as working hours, the number of night shifts, and the number of days off among healthcare workers in Japan: a cross-sectional study

**DOI:** 10.1186/s12913-020-05114-8

**Published:** 2020-04-15

**Authors:** Ryosuke Hayashi, Shigeru Fujita, Shuhei Iida, Yoji Nagai, Yoshiko Shimamori, Tomonori Hasegawa

**Affiliations:** 1 Toho University Graduate School of Medicine, Tokyo, Japan; 2 Department of Social Medicine, Toho University School of Medicine, Tokyo, Japan; 3 Nerima General Hospital, Tokyo, Japan; 4 Institute of Healthcare Quality Improvement, Tokyo, Japan; 5 Hitachinaka General Hospital, Ibaraki, Japan; 6 Department of Common Fundamental Nursing, Iwate Medical University School of Nursing, Iwate, Japan

**Keywords:** Patient safety, Safety management, Patient safety culture, Workload, Fatigue

## Abstract

**Background:**

Patient safety culture is defined as a product of individual and group values, attitudes, perceptions, competencies, and patterns of behavior that determine the commitment to, and the style and proficiency of, an organization’s health and safety management. Factors influencing healthcare workers’ working environment such as working hours, the number of night shifts, and the number of days off may be associated with patient safety culture, and the association pattern may differ by profession. This study aimed to examine the relationship between patient safety culture and working environment.

**Methods:**

Questionnaire surveys were conducted in 2015 and 2016. The first survey was conducted in hospitals in Japan to investigate their patient safety management system and activities and intention to participate in the second survey. The second survey was conducted in 40 hospitals; 100 healthcare workers from each hospital answered a questionnaire that was the Japanese version of the Hospital Survey on Patient Safety Culture for measuring patient safety culture. The relationship of patient safety culture with working hours in a week, the number of night shifts in a month, and the number of days off in a month was analyzed.

**Results:**

Response rates for the first and second surveys were 22.4% (731/3270) and 94.2% (3768/4000), respectively. Long working hours, numerous night shifts, and few days off were associated with low patient safety culture. Despite adjusting the working hours, the number of event reports increased with an increase in the number of night shifts. Physicians worked longer and had fewer days off than nurses. However, physicians had fewer composites of patient safety culture score related to working hours, the number of night shifts, and the number of days off than nurses.

**Conclusions:**

This study suggested a possibility of improving the patient safety culture by managing the working environment of healthcare workers. High number of night shifts may lead to high number of event reports. Working hours, the number of night shifts, and the number of days off may differently influence patient safety culture in physicians and nurses.

## Background

In a report published in 1999, the Institute of Medicine advocated establishment of a system that promotes patient safety and advised that medical institutions should develop a safety culture to prevent adverse events [1]. Patient safety culture is a product of individual and group values, attitudes, perceptions, competencies, and behavior patterns that determines the commitment as well as the style and proficiency of an organization’s health and safety management [2]. Patient safety education and training [3, 4] as well as utilization of Team Strategies and Tools to Enhance Performance and Patient Safety [5] and root cause analysis [6] are useful for improving patient safety culture. We have reported that long working hours of nurses may deteriorate their patient safety culture [7], and two studies in China and a study in Korea have also reported same results [8–10]. Some studies have suggested that working environment deterioration of healthcare workers is associated with an increase in the number of event reports [11, 12]; however, few studies have examined the influence of working hours, the number of night shifts, and the number of days off on patient safety culture. Working environment associated with each profession may affect patient safety culture differently because each profession has different working environment characteristics [13–15].

This study aimed to investigate the relationship of patient safety culture of healthcare workers with their working hours, the number of night shifts, and the number of days off and to clarify the differences in this relationship depending on the profession.

## Methods

In this study, two questionnaire surveys were conducted in 2015 and 2016. The first survey aimed to clarify hospitals’ patient safety management system and activities as well as their intention to participate in the second survey. From August to September 2015, an anonymous nationwide mail survey was conducted in several hospitals in Japan. Among all hospitals in Japan, 25.0, 50.0, and 100% hospitals with < 100 beds, 100–299 beds, and ≥ 300 beds, respectively, were randomly selected as target hospitals because patient safety management system or activities in each hospital may vary depending on the hospital size. University affiliated hospitals were excluded because they are obligated to assign full-time patient safety managers including a physician, a nurse, and a pharmacist, but others are not obligated to assign them. Respondents of the first survey were chief medical directors or patient safety managers in the target hospitals.

The second survey, conducted in 2016, aimed to measure patient safety culture of healthcare workers. Among the hospitals that expressed their intention to participate in the second survey, acute care hospitals with ≥300 beds using an electronic medical record system were selected as target hospitals. The questionnaires were distributed to 100 healthcare workers in each target hospital. The healthcare workers comprised 12 physicians, 66 nurses, 16 technicians/therapists, and 6 pharmacists. The number of professions was determined according to the number of professions in the national statistics of healthcare workers in Japanese hospitals [16]. If the number of those professions was insufficient, each hospital was permitted to include other professions, such as a dietitian, cook, or clerk. A total of 100 healthcare workers were included; they were selected by patient safety managers in each hospital using the purposive selection method. Random sampling was not used to reduce the effort of counterpart at each hospital. To avoid bias in selecting respondents with a specific patient safety culture, patient safety managers were requested to distribute the questionnaires to healthcare workers without bias in the number of years of experience, position, and involvement in patient safety activities and to distribute them to nurses in multiple wards, including both internal medicine and surgical wards.

### Measurement

The first survey questionnaire included questions on the functions of the hospital, the number of beds, and the assignment status of patient safety managers and the intention to participate in the second survey.

The second survey questionnaire included questions on respondents’ working hours in a week, the number of night shifts in a month, the number of days off in a month, and patient safety culture. As for the response options, Likert scales were used for those questions. The patient safety culture was measured by the Japanese version of the Hospital Survey on Patient Safety Culture developed by the United States Agency for Healthcare Research and Quality [2, 17]. This survey comprises 44 questions and can calculate scores for 12 composites related to patient safety culture as well as scores for patient safety grade and number of events reported in two items. For those questions, Likert scales with 5-point response options for agreement (1: Strongly disagree to 5: Strongly agree), frequency (1: Never to 5: Always), Patient Safety Grade (1: Failing to 5: Excellent), and number of events reported (from 1: No events to 5: 21 events or more) were used. The 12 composite scores were calculated according to a guideline of the Agency for Healthcare Research and Quality [18]. Proportion of positive responses for those questions is calculated as a composite score. The second survey questionnaire is shown in Additional file 2.

A higher score means a better patient safety culture. Regarding patient safety grade, the respondents were classified into two groups: those who answered “very good” or “good” and the others. Regarding the number of events reported, the respondents were also classified into two groups: those who reported one or more adverse events or near misses and the others. Regarding the working hours in a week, the number of night shifts, and the number of days off, the respondents were classified into three groups as shown in Table 1.

**Table 1:** Characteristics of respondents

		All respondents		Nurses		Physicians		Others	
		n	(%)	n	(%)	n	(%)	n	(%)
**Years of experience**	< 1	203	(5.4)	143	(6.0)	13	(3.5)	47	(4.6)
	1–5	986	(26.2)	680	(28.5)	66	(17.9)	240	(23.7)
	6–10	631	(16.7)	399	(16.7)	51	(13.9)	181	(17.9)
	11–15	551	(14.6)	358	(15.0)	48	(13.0)	145	(14.3)
	16–20	467	(12.4)	296	(12.4)	42	(11.4)	129	(12.7)
	≥21	802	(21.3)	428	(17.9)	137	(37.2)	237	(23.4)
	No answer	128	(3.4)	83	(3.5)	11	(3.0)	34	(3.4)
**Working hours in a week**	< 40	1154	(30.6)	872	(36.5)	13	(3.5)	269	(26.6)
	40–60	2145	(56.9)	1392	(58.3)	191	(51.9)	652	(64.4)
	≥60	365	(9.7)	130	(5.4)	162	(44.0)	73	(7.2)
	No answer	104	(2.8)	83	(3.5)	2	(0.5)	19	(1.9)
**Number of night shifts in a month**	0	764	(20.3)	243	(10.2)	86	(23.4)	435	(42.9)
	1–4	1628	(43.2)	888	(37.2)	225	(61.1)	515	(50.8)
	≥5	1347	(35.7)	1239	(51.9)	55	(14.9)	53	(5.2)
	No answer	29	(0.8)	17	(0.7)	2	(0.5)	10	(1.0)
**Number of days off in a month**	≥10	1049	(27.8)	875	(36.7)	4	(1.1)	170	(16.8)
	7–9	2137	(56.7)	1359	(56.9)	106	(28.8)	672	(66.3)
	< 7	487	(12.9)	83	(3.5)	253	(68.8)	151	(14.9)
	No answer	95	(2.5)	70	(2.9)	5	(1.4)	20	(2.0)
**Total**		3768	(100.0)	2387	(100.0)	368	(100.0)	1013	(100.0)

### Data analysis

Patient safety culture scores for all respondents were calculated, and the relationship of these scores with working hours in a week, the number of night shifts in a month, and the number of days off in a month was analyzed.

The relationship of composite scores with working hours in a week, the number of night shifts in a month, and the number of days off in a month was analyzed using Spearman’s ρ. The relationship of the patient safety grade and the number of events reported with working hours in a week, the number of night shifts in a month, and the number of days off in a month was analyzed using Cramer’s V.

To analyze the relationship of patient safety culture scores with working hours in a week, the number of night shifts in a month, and the number of days off in a month, a generalized linear mixed model (GLMM) was used. The GLMM analysis was performed for all respondents, for physicians, and for nurses. Furthermore, regarding GLMM, the targets were patient safety culture scores; the fixed effects were working hours in a week, the number of night shifts in a month, and the number of days off in a month; and the random effect was the difference of hospitals. In the analysis of all respondents, the results were adjusted by profession and the years of experience. In the analysis of each profession, the results were adjusted by the years of experience. In the analysis for physicians, the working hours in a week and the number of days off in a month were classified into two groups because the sample size of light workload group was too small to analyze. *P* < 0.05 indicated significance. Missing data were excluded from the analysis. IBM SPSS Statistics version 19 was used for statistical analysis.

### Ethical considerations

This study was approved by the Ethics Committee of the Toho University School of Medicine (No. 27045).

## Results

The response rate for the first survey was 22.4% (731/3270). The response rates varied by hospital size: that in hospitals with 300 beds or more was 24.1%, that in hospitals with 100–299 beds was 21.4%, and that in hospitals with less than 100 beds was 13.9%. Among the participating hospitals, 205 responded that they intended to participate in the second survey; however, only 81 hospitals fulfilled the criteria for the second survey. For these 81 hospitals, we confirmed the intention to participate in the second survey again because the second survey was conducted approximately 1 year after the first survey, and consequently, 40 hospitals participated.

The second survey was conducted in these 40 hospitals in which 9 are located in urban areas and others are located in rural areas. Among them, 14 hospitals have 300–399 beds, 13 hospitals have 400–499 beds, and 13 hospitals have 500 beds or more. The response rate for the second survey was 94.2% (3768/4000). Among the respondents, 66.7% were nurses and nursing aids, 9.8% were physicians, 6.1% were pharmacists, 15.4% were therapists and technicians, and 1.9% were the others. The proportion of each profession corresponded to the number of distributions by profession.

Characteristics of the respondents are shown in Table 1. The proportion of physicians who worked ≥60 h in a week was higher than that of nurses (44.3% vs. 5.6%, *P* < 0.01); furthermore, the proportion of physicians who had less than 7 days off in a month was also higher than that of nurses (69.7% vs. 3.6%, *P* < 0.01).

Table 2 and Table A1 shown in Additional file 1 presents the mean patient safety culture scores and their correlation with working hours, the number of night shifts, and the number of days off. The scores of eight composites tended to decrease as the working hours increased although the correlation coefficients represent small associations. Similarly, the scores of seven composites tended to decrease as the number of night shifts increased. Furthermore, the scores of eight composites tended to decrease as the number of days off decreased.

**Table 2:** Patient safety culture scores in all respondents

	All respondents	Working hours in a week				Number of night shifts in a month				Number of days off in a month			
		< 40	40–60	≥60	Spearman’s ρ	0	1–4	≥5	Spearman’s ρ	≥10	7–9	< 7	Spearman’s ρ
**(Number of respondents)**	(3768)	(1154)	(2145)	(365)		(764)	(1628)	(1347)		(1049)	(2137)	(487)	
**Teamwork within units**	0.76	0.79	0.75	0.73	−0.07^a^	0.76	0.76	0.76	0.01	0.80	0.75	0.72	−0.08^a^
**Supervisor or manager expectations and actions promoting patient safety**	0.70	0.72	0.69	0.68	−0.06^a^	0.68	0.71	0.70	0.03	0.73	0.69	0.66	−0.07^a^
**Organizational learning-continuous improvement**	0.58	0.61	0.57	0.57	− 0.05^a^	0.61	0.59	0.56	−0.05^a^	0.61	0.57	0.55	−0.07^a^
**Management support for patient safety**	0.61	0.64	0.60	0.54	−0.08^a^	0.62	0.62	0.58	−0.04^a^	0.65	0.60	0.56	−0.08^a^
**Overall perceptions of patient safety**	0.59	0.61	0.58	0.54	−0.06^a^	0.63	0.60	0.55	−0.09^a^	0.59	0.59	0.58	0.00
**Feedback and communication about error**	0.66	0.70	0.66	0.53	−0.11^a^	0.65	0.64	0.68	0.05^b^	0.72	0.66	0.52	−0.15^a^
**Communication openness**	0.57	0.59	0.57	0.56	−0.03	0.58	0.58	0.56	−0.02	0.60	0.57	0.56	−0.04^b^
**Frequency of events reported**	0.71	0.71	0.72	0.65	−0.02	0.70	0.71	0.72	0.02	0.72	0.71	0.64	−0.04^b^
**Teamwork across units**	0.49	0.51	0.48	0.51	−0.03	0.50	0.51	0.47	−0.05^a^	0.51	0.48	0.52	0.00
**Staffing**	0.42	0.46	0.41	0.37	−0.09^a^	0.46	0.45	0.37	−0.14^a^	0.42	0.43	0.41	0.00
**Handoffs and transitions**	0.35	0.37	0.35	0.28	−0.07^a^	0.36	0.36	0.34	−0.01	0.37	0.34	0.32	−0.05^a^
**Nonpunitive response to errors**	0.51	0.52	0.50	0.49	−0.03	0.55	0.54	0.45	−0.11^a^	0.49	0.51	0.52	0.02
**Proportion of respondents who rated the patient safety grade as “very good” or “good”**	0.48	0.52	0.47	0.43	0.06^a,c^	0.50	0.50	0.44	0.06^a,c^	0.47	0.48	0.50	0.02^c^
**Proportion of respondents who reported one or more events**	0.79	0.79	0.80	0.70	0.07^a,c^	0.60	0.79	0.90	0.26^a,c^	0.84	0.80	0.64	0.16^a,c^

^a^*P* < 0.01

^b^*P* < 0.05

^c^Cramer’s V

The GLMM analysis results for all respondents are shown in Table 3. Patient safety culture composite scores tended to decrease as working hours and the number of night shifts increased or the number of days off decreased. Tables 4 and 5 present the GLMM analysis results for nurses and physicians, respectively. Regarding nurses, working hours were associated with the scores of seven composites and patient safety grade, the number of night shifts was associated with the scores of three composites and the number of events reported, and the number of days off was associated with the scores of seven composites. Regarding physicians, working hours were associated with the scores of two composites, the number of night shifts was associated with the scores of three composites and patient safety grade, and the number of days off was associated with the score of one composite.

**Table 3:** Relationship between the working environment and patient safety culture scores in all respondents^a^

Response variables		Explanatory variables									AICC
		Working hours in a week			Number of night shifts in a month			Number of days off in a month			
		< 40	40–60	≥60	0	1–4	≥5	≥10	7–9	< 7	
	(n)	(1154)	(2145)	(365)	(764)	(1628)	(1347)	(1049)	(2137)	(487)	
**Teamwork within units**	Coefficient	0.00	−0.04^b^	− 0.08^c^	0.00	−0.03^c^	−0.06^c^	0.00	−0.04^c^	−0.10^c^	1738
**Supervisor or manager expectations and actions promoting patient safety**		0.00	−0.02^c^	−0.01	0.00	0.00	−0.03	0.00	−0.03^b^	−0.06^b^	1345
**Organizational learning-continuous improvement**		0.00	− 0.03^c^	0.00	0.00	−0.04^c^	−0.08^c^	0.00	−0.03^c^	−0.02	2286
**Management support for patient safety**		0.00	−0.03^c^	−0.11^c^	0.00	−0.01	−0.06	0.00	−0.05^b^	−0.08^b^	2640
**Overall perceptions of patient safety**		0.00	−0.04^b^	−0.09^b^	0.00	−0.04^c^	−0.07^c^	0.00	−0.01	−0.03	2268
**Feedback and communication about error**		0.00	−0.01	− 0.05^c^	0.00	−0.04^c^	−0.05^b^	0.00	− 0.04^b^	−0.07^b^	2599
**Communication openness**		0.00	−0.02	− 0.03	0.00	− 0.04^c^	−0.07^b^	0.00	− 0.03^c^	−0.07^b^	2764
**Frequency of events reported**		0.00	0.02	0.02	0.00	−0.02	− 0.02	0.00	−0.01	−0.02	2903
**Teamwork across units**		0.00	−0.05^b^	−0.05^c^	0.00	−0.01	−0.03	0.00	−0.04^c^	−0.06^c^	2404
**Staffing**		0.00	−0.05^b^	−0.12 ^b^	0.00	0.02	−0.04 ^b^	0.00	−0.02	−0.09^b^	908
**Handoffs and transitions**		0.00	− 0.02	− 0.08^b^	0.00	0.01	0.00	0.00	−0.03	−0.05^c^	2282
**Nonpunitive response to errors**		0.00	−0.04^c^	− 0.08^b^	0.00	−0.01	−0.08^b^	0.00	−0.01	−0.08^b^	2967
**Respondents who rated patient safety grade as “very good” or “good” (vs. others)**	aOR	1.00	0.73^b^	0.56^b^	1.00	0.88	0.76^c^	1.00	1.02	1.05	14,247
**Respondents who reported one or more events (vs. none)**		1.00	1.33^c^	1.29	1.00	1.72^b^	2.66^b^	1.00	0.96	0.90	17,215

*AICC* Akaike’s Information Criterion Correction, *aOR*: adjusted Odds Ratio, *(n)*: Number of respondents

^a^Results of the generalized linear mixed model using working hours, the number of night shifts, and the number of days off as explanatory variables

^b^*P* < 0.01

^c^*P* < 0.05

**Table 4:** Relationship between the working environment and patient safety culture scores in nurses^a^

Response variables		Explanatory variables									AICC
		Working hours in a week			Number of night shifts in a month			Number of days off in a month			
		< 40	40–60	≥60	0	1–4	≥5	≥10	7–9	< 7	
	(n)	(872)	(1392)	(130)	(243)	(888)	(1239)	(875)	(1359)	(83)	
**Teamwork within units**	Coefficient	0.00	−0.04^b^	−0.07^c^	0.00	0.02	− 0.03	0.00	−0.04^b^	− 0.07^c^	932
**Supervisor or manager expectations and actions promoting patient safety**		0.00	−0.01	0.00	0.00	0.00	−0.03	0.00	−0.05^b^	−0.12^b^	721
**Organizational learning-continuous improvement**		0.00	−0.04^c^	0.02	0.00	−0.04	−0.09^b^	0.00	−0.03^c^	−0.02	1444
**Management support for patient safety**		0.00	−0.02	− 0.05	0.00	0.00	−0.05	0.00	−0.05^b^	− 0.06	1632
**Overall perceptions of patient safety**		0.00	−0.03	− 0.11^b^	0.00	−0.05^c^	−0.08^b^	0.00	−0.01	−0.03	1401
**Feedback and communication about error**		0.00	−0.02	− 0.04	0.00	−0.04	−0.04	0.00	−0.02	−0.05	1468
**Communication openness**		0.00	−0.02	0.01	0.00	−0.02	−0.04	0.00	−0.03	−0.06	1735
**Frequency of events reported**		0.00	0.02	0.02	0.00	−0.01	− 0.01	0.00	−0.01	0.01	1721
**Teamwork across units**		0.00	−0.02	−0.07^c^	0.00	−0.01	− 0.05	0.00	−0.04^b^	− 0.06	1469
**Staffing**		0.00	−0.05^b^	−0.14^b^	0.00	−0.01	− 0.06^b^	0.00	−0.02	−0.07^c^	458
**Handoffs and transitions**		0.00	0.00	−0.08^c^	0.00	0.01	−0.01	0.00	−0.02	−0.05	1429
**Nonpunitive response to errors**		0.00	−0.03	− 0.09^b^	0.00	0.05	−0.04	0.00	−0.02	−0.12^b^	1829
**Respondents who rated patient safety grade as “very good” or “good” (vs. others)**	aOR	1.00	0.73^b^	0.69	1.00	0.87	0.73	1.00	1.05	1.09	8877
**Respondents who reported one or more events (vs. none)**		1.00	1.02	1.08	1.00	2.28^b^	3.58^b^	1.00	1.13	1.00	11,529

*AICC* Akaike’s Information Criterion Correction, *aOR*: adjusted Odds Ratio, (*n*) Number of respondents

^a^Results of the generalized linear mixed model using working hours, the number of night shifts and the number of days off as explanatory variables

^b^*P* < 0.01

^c^*P* < 0.05

**Table 5:** Relationship between the working environment and patient safety culture scores in physicians^a^

Response variables		Explanatory variables							AICC
		Working hours in a week		Number of night shifts in a month			Number of days off in a month		
		< 60	≥60	0	1–4	≥5	≥7	≤6	
	(n)	(204)	(162)	(86)	(225)	(55)	(110)	(253)	
**Teamwork within units**	Coefficient	0.00	−0.08^b^	0.00	−0.11^c^	− 0.07	0.00	− 0.05	140
**Supervisor or manager expectations and actions promoting patient safety**		0.00	0.01	0.00	0.01	−0.03	0.00	0.03	203
**Organizational learning-continuous improvement**		0.00	−0.02	0.00	−0.05	−0.08	0.00	−0.02	259
**Management support for patient safety**		0.00	−0.13^c^	0.00	−0.04	−0.06	0.00	−0.07	301
**Overall perceptions of patient safety**		0.00	−0.04	0.00	−0.04	−0.03	0.00	−0.06	244
**Feedback and communication about error**		0.00	−0.07	0.00	−0.15^c^	−0.23^c^	0.00	−0.05	339
**Communication openness**		0.00	−0.05	0.00	−0.09	−0.13	0.00	−0.01	316
**Frequency of events reported**		0.00	0.02	0.00	−0.14^b^	−0.23^c^	0.00	−0.05	377
**Teamwork across units**		0.00	0.02	0.00	−0.03	−0.03	0.00	0.02	301
**Staffing**		0.00	−0.06	0.00	0.07	0.05	0.00	−0.12^c^	137
**Handoffs and transitions**		0.00	− 0.05	0.00	0.08	0.02	0.00	−0.06	265
**Nonpunitive response to errors**		0.00	−0.02	0.00	0.02	−0.02	0.00	−0.03	309
**Respondents who rated patient safety grade as “very good” or “good” (vs. others)**	aOR	1.00	0.55^b^	1.00	0.57	0.40^b^	1.00	0.75	1464
**Respondents who reported one or more events (vs. none)**		1.00	1.45	1.00	1.38	1.76	1.00	1.44	1500

The number of physicians who worked less than 40 h or had 10 days off or more was few, and working hours and the number of days off were divided into two groups

*AICC* Akaike’s Information Criterion Correction, *aOR* adjusted Odds Ratio, (*n*) Number of respondents

^a^Results of the generalized linear mixed model using working hours, the number of night shifts, and the number of days off as explanatory variables

^b^*P* < 0.05

^c^*P* < 0.01

## Discussion

The study results suggest that not only working hours but also the number of night shifts and days off of healthcare workers are related to patient safety culture. Therefore, these factors should be managed cautiously to improve patient safety culture.

Previous studies in China reported that long working hours may deteriorate patient safety culture of healthcare workers [8, 9]. In Korea, dental hygienists who worked more than 40 h in a week showed lower scores in most composites excepting “organizational learning-continuous improvement” than those who worked 40 h or less in a week [10]. Regarding the relationship of working hours with patient safety culture, previous studies show same tendency with our results [7–10], but no study shows the relationship of number of night shifts and days off with patient safety culture.

Regarding the analysis for all respondents, the number of event reports increased as the number of night shifts increased, despite adjusting working hours. Considering that the composite score of “frequency of events reported” was not related to working hours and the number of night shifts, the increase in the number of night shifts could lead to an increase in adverse events or near misses.

Physicians’ workload was higher than that of nurses; however, the number of composites associated with working hours, the number of night shifts, and the number of days off for physicians was fewer than that for nurses. This difference between physicians and nurses suggests that different mechanisms or factors should be considered for determining the relationship between workload and patient safety culture of these professions. Heavy workload increases the occupational stress, anxiety, and depression among healthcare workers [19]. In addition, the increasing occupational stress, anxiety, and depression increase the number of adverse events and near misses [11, 12, 20, 21]. Patient safety culture may be similarly affected by occupational stress, anxiety, and depression, which are, in turn, influenced by working hours, the number of night shifts, and the number of days off. Physician’s stress response for occupational stress was reportedly weaker than that of nurses because physicians got more supports from their supervisors and colleagues than nurses, and physicians have more discretionary power than nurses [22]. In addition, nurses tend to have stress responses because they spend a lot of times for direct interaction or contact with patients, and work physically demanding [22]. In our study, physicians’ workload was higher than that of nurses, but the stress response of physicians may have been weaker than that of nurses. Consequently, composites associated with working hours, the number of night shifts, and the number of days off in physicians may be less than those in nurses.

Regarding nurses, working hours, the number of night shifts, and the number of days off were not associated with the scores in two composites: “feedback and communication about error” and “frequency of events reported.” The attitudes of nurses toward event reporting and error discussion might be affected by other factors such as the implementation of a simple and easy event reporting system and good teamwork within units [23–26]. Meanwhile, physicians’ scores for these two composites decreased as the number of night shifts increased. Physicians might stop reporting events and discussing about errors when the number of night shifts increases; however, the reason for this is unknown. Hence, the underlying reasons need to be determined in the future.

### Limitations of this study

This was a cross-sectional study; thus, the causal relationship remains unclear. The response rate for the first survey was not high especially in small hospitals, and the situations in small hospitals may not be fully reflected in our results because acute care hospitals with < 300 beds or long-term care hospitals were not included in the second survey. Agency for Healthcare Research and Quality reported that patient safety culture scores in small hospitals were higher than those in large hospitals [2]. The impact of the working environment in small hospitals on the patient safety culture can be different from that in large hospitals. In addition, the responding facilities could be the hospitals with a good patient safety culture. In the second survey, patient safety managers in each hospital selected the respondents using purposive selection method; these selected respondents might already have been highly aware of patient safety. It was unknown whether the respondents were selected without bias at each hospital. The next study may need to use random sampling to select healthcare workers at each hospital. However, the influences of working hours, the number of night shifts, and the number of days off on patient safety culture might be similar among healthcare workers with better or worse patient safety culture. In this study, each hospital was permitted to include other professions, such as a dietitian, cook, or clerk if the number of designated professions was insufficient. However, those respondents accounted for only 0.9%, and those respondents may have little effect on the results. This study did not aim to figure out patient safety culture of the target population but to determine the relationship of patient safety culture with working hours, the number of night shifts, and the number of days off; the limitation of representativeness in our study could have minimal effects on the results. Regarding the GLMMs, up to 9.1% of the data was excluded from the analysis due to the rate of missing answers to questions, and the missing data may have little effect on the results.

## Conclusion

This study suggested a possibility of improving the patient safety culture by managing the working environment of healthcare workers. Proper management of the number of night shifts and days off as well as working hours of healthcare workers might improve patient safety culture. An increase in the number of night shifts might lead to an increase in the reports of adverse events and near misses. Working hours, the number of night shifts, and the number of days off differently influence patient safety culture of physicians and nurses.

## Supplementary information

**Additional file 1.** Table A1. Patient Safety Culture Scores and SD in all Respondents.

**Additional file 2.** A survey for working environment and patient safety culture.

## Data Availability

All data supporting our findings were presented within the manuscript.

## Abbreviations

GLMM: generalized linear mixed model
AICC: Akaike’s Information Criterion Correction
aOR: adjusted Odds Ratio
SD: Standard Deviation
