# Supplementary material for: Relationship of patient safety culture with factors influencing working environment such as working hours, the number of night shifts, and the number of days off among healthcare workers in Japan: a cross-sectional study
Source: BMC Health Serv Res. 2020 Apr 15;20:310. doi: 10.1186/s12913-020-05114-8 (PMC7158118; doi:10.1186/s12913-020-05114-8)
Supplement: Supplementary file 2 — Additional file 2. A survey for working environment and patient safety culture. [file 12913_2020_5114_MOESM2_ESM.docx]

A survey for working environment and patient safety culture

**I. Working environment**

| 1 | Department | □1 Physician’s department ( ) □2 Outpatient department  □3 Internal medicine ward □4 Surgical ward  □5 Mixed ward of internal medicine and surgery □6 Other wards ( ) □7 Test and examination department | | | □8 Operation department □9 Critical care center or intensive care unit □10 Pharmaceutical department □11 Rehabilitation department □12 Food and nutrition department or cafeteria □13 Administration department □14 Others ( ) | |
| --- | --- | --- | --- | --- | --- | --- |
| 2 | Profession | □1 Nurse □2 Nursing aid □3 Physician □4 Resident | □5 Pharmacist  □6 Dietician or cook □7 Therapist  □8 Technician | | | □ 9 Clerk □10 Others ( ) |
| 3 | Working hours in a week | | | <20 h ・ 20–40 h ・ 40–60 h 60–80 h ・ 80–100 h ・ ≥100 h | | |
| 4 | Number of nightshifts in a month | | | 0 ・ 1–4 ・ 5–8 ・ 9–12 ・ ≥13 | | |
| 5 | Number of days off in a month | | | <4 days ・ 4–6 days ・ 7–9 days ・ ≥10 days | | |
| 6 | Do you think your hospital is focused on staff education? | | | Yes ・ No ・ Neither | | |
| 7 | Have you participated in-hospital patient safety workshops within the past year? | | | Did not participated ・Participated once ・Participated more than once | | |
| 8 | Would you like to encourage your friends or relatives to consult your hospital? | | | Yes ・ No ・ Neither | | |

**II. Patient Safety Culture**

This survey asks for your opinions about patient safety issues, medical error, and event reporting in your hospital

<Definitions>

1. An “event” is defined as any type of error, mistake, incident, accident, or deviation, regardless of whether or not it results in patient harm.
2. “Patient safety” is defined as the avoidance and prevention of patient injuries or adverse events resulting from the processes of health care delivery.
3. In this survey, think of your “unit” as the work area, department, or clinical area of the hospital where you spend most of your work time or provide most of your clinical services.

**SECTION A: Your Work Area/Unit**

**Please indicate your agreement or disagreement with the following statements about your work area/unit.**

| **Think about your hospital work area/unit…** | **Strongly Disagree** ⯆ | **Disagree** ⯆ | **Neither** ⯆ | **Agree** ⯆ | **Strongly Agree** ⯆ |
| --- | --- | --- | --- | --- | --- |
| 9. People support one another in this unit | 🞎1 | 🞎2 | 🞏3 | 🞎4 | 🞏5 |
| 10. We have enough staff to handle the workload | 🞎1 | 🞎2 | 🞏3 | 🞎4 | 🞏5 |
| 11. When a lot of work needs to be done quickly, we work together as a team to get the work done | 🞎1 | 🞎2 | 🞏3 | 🞎4 | 🞏5 |
| 12. In this unit, people treat each other with respect | 🞎1 | 🞎2 | 🞏3 | 🞎4 | 🞏5 |
| 13. Staff in this unit work longer hours than is best for patient care | 🞎1 | 🞎2 | 🞏3 | 🞎4 | 🞏5 |

**SECTION A: Your Work Area/Unit (continued)**

| **Think about your hospital work area/unit…** | **Strongly Disagree** ⯆ | **Disagree** ⯆ | **Neither** ⯆ | **Agree** ⯆ | **Strongly Agree** ⯆ |
| --- | --- | --- | --- | --- | --- |
| 14. We are actively doing things to improve patient safety | 🞎1 | 🞎2 | 🞏3 | 🞎4 | 🞏5 |
| 15. We use more agency/temporary staff than is best for patient care | 🞎1 | 🞎2 | 🞏3 | 🞎4 | 🞏5 |
| 16. Staff feel like their mistakes are held against them | 🞎1 | 🞎2 | 🞏3 | 🞎4 | 🞏5 |
| 17. Mistakes have led to positive changes here | 🞎1 | 🞎2 | 🞏3 | 🞎4 | 🞏5 |
| 18. It is just by chance that more serious mistakes don’t happen around here | 🞎1 | 🞎2 | 🞏3 | 🞎4 | 🞏5 |
| 19. When one area in this unit gets really busy, others help out | 🞎1 | 🞎2 | 🞏3 | 🞎4 | 🞏5 |
| 20. When an event is reported, it feels like the person is being written up, not the problem | 🞎1 | 🞎2 | 🞏3 | 🞎4 | 🞏5 |
| 21. After we make changes to improve patient safety, we evaluate their effectiveness | 🞎1 | 🞎2 | 🞏3 | 🞎4 | 🞏5 |
| 22. We work in "crisis mode" trying to do too much, too quickly | 🞎1 | 🞎2 | 🞏3 | 🞎4 | 🞏5 |
| 23. Patient safety is never sacrificed to get more work done | 🞎1 | 🞎2 | 🞏3 | 🞎4 | 🞏5 |
| 24. Staff worry that mistakes they make are kept in their personnel file | 🞎1 | 🞎2 | 🞏3 | 🞎4 | 🞏5 |
| 25. We have patient safety problems in this unit | 🞎1 | 🞎2 | 🞏3 | 🞎4 | 🞏5 |
| 26. Our procedures and systems are good at preventing errors from happening | 🞎1 | 🞎2 | 🞏3 | 🞎4 | 🞏5 |

**SECTION B: Your Supervisor/Manager**

**Please indicate your agreement or disagreement with the following statements about your immediate supervisor/manager or person to whom you directly report.**

|  | **Strongly Disagree** ⯆ | **Disagree** ⯆ | **Neither** ⯆ | **Agree** ⯆ | **Strongly Agree** ⯆ |
| --- | --- | --- | --- | --- | --- |
| 27. My supervisor/manager says a good word when he/she sees a job done according to established patient safety procedures | 🞎1 | 🞎2 | 🞏3 | 🞎4 | 🞏5 |
| 28. My supervisor/manager seriously considers staff suggestions for improving patient safety | 🞎1 | 🞎2 | 🞏3 | 🞎4 | 🞏5 |
| 29. Whenever pressure builds up, my supervisor/manager wants us to work faster, even if it means taking shortcuts | 🞎1 | 🞎2 | 🞏3 | 🞎4 | 🞏5 |
| 30. My supervisor/manager overlooks patient safety problems that happen over and over | 🞎1 | 🞎2 | 🞏3 | 🞎4 | 🞏5 |

**SECTION C: Communications**

**How often do the following things happen in your work area/unit?**

| **Think about your hospital work area/unit…** | **Never** ⯆ | **Rarely** ⯆ | **Some-times** ⯆ | **Most of the time** ⯆ | **Always** ⯆ |
| --- | --- | --- | --- | --- | --- |
| 31. We are given feedback about changes put into place based on event reports | 🞎1 | 🞎2 | 🞏3 | 🞎4 | 🞏5 |
| 32. Staff will freely speak up if they see something that may negatively affect patient care | 🞎1 | 🞎2 | 🞏3 | 🞎4 | 🞏5 |
| 33. We are informed about errors that happen in this unit | 🞎1 | 🞎2 | 🞏3 | 🞎4 | 🞏5 |
| 34. Staff feel free to question the decisions or actions of those with more authority | 🞎1 | 🞎2 | 🞏3 | 🞎4 | 🞏5 |
| 35. In this unit, we discuss ways to prevent errors from happening again | 🞎1 | 🞎2 | 🞏3 | 🞎4 | 🞏5 |
| 36. Staff are afraid to ask questions when something does not seem right | 🞎1 | 🞎2 | 🞏3 | 🞎4 | 🞏5 |

**SECTION D: Frequency of Events Reported**

**In your hospital work area/unit, when the following mistakes happen, *how often are they reported?***

|  | **Never** ⯆ | **Rarely** ⯆ | **Some-times** ⯆ | **Most of the time** ⯆ | **Always** ⯆ |
| --- | --- | --- | --- | --- | --- |
| 37. When a mistake is made, but is *caught and corrected before affecting the patient*, how often is this reported? | 🞎1 | 🞎2 | 🞏3 | 🞎4 | 🞏5 |
| 38. When a mistake is made, but has *no potential to harm the patient*, how often is this reported? | 🞎1 | 🞎2 | 🞏3 | 🞎4 | 🞏5 |
| 39. When a mistake is made that *could harm the patient*, but does not, how often is this reported? | 🞎1 | 🞎2 | 🞏3 | 🞎4 | 🞏5 |

**SECTION E: Patient Safety Grade**

40. **Please give your work area/unit in this hospital an overall grade on patient safety.**

| 🞎 | 🞎 | 🞎 | 🞎 | 🞎 |
| --- | --- | --- | --- | --- |
| **A**  Excellent | **B**  Very Good | **C**  Acceptable | **D**  Poor | **E**  Failing |

**SECTION F: Your Hospital**

**Please indicate your agreement or disagreement with the following statements about your hospital.**

| **Think about your hospital…** | **Strongly Disagree** ⯆ | **Disagree** ⯆ | **Neither** ⯆ | **Agree** ⯆ | **Strongly Agree** ⯆ |
| --- | --- | --- | --- | --- | --- |
| 41. Hospital management provides a work climate that promotes patient safety | 🞎1 | 🞎2 | 🞏3 | 🞎4 | 🞏5 |
| 42. Hospital units do not coordinate well with each other | 🞎1 | 🞎2 | 🞏3 | 🞎4 | 🞏5 |
| 43. Things “fall between the cracks” when transferring patients from one unit to another | 🞎1 | 🞎2 | 🞏3 | 🞎4 | 🞏5 |
| 44. There is good cooperation among hospital units that need to work together | 🞎1 | 🞎2 | 🞏3 | 🞎4 | 🞏5 |
| **SECTION F: Your Hospital (continued)** |  |  |  |  |  |
| **Think about your hospital…** | **Strongly Disagree** ⯆ | **Disagree** ⯆ | **Neither** ⯆ | **Agree** ⯆ | **Strongly Agree** ⯆ |
| 45. Important patient care information is often lost during shift changes | 🞎1 | 🞎2 | 🞏3 | 🞎4 | 🞏5 |
| 46. It is often unpleasant to work with staff from other hospital units | 🞎1 | 🞎2 | 🞏3 | 🞎4 | 🞏5 |
| 47. Problems often occur in the exchange of information across hospital units | 🞎1 | 🞎2 | 🞏3 | 🞎4 | 🞏5 |
| 48. The actions of hospital management show that patient safety is a top priority | 🞎1 | 🞎2 | 🞏3 | 🞎4 | 🞏5 |
| 49. Hospital management seems interested in patient safety only after an adverse event happens | 🞎1 | 🞎2 | 🞏3 | 🞎4 | 🞏5 |
| 50. Hospital units work well together to provide the best care for patients | 🞎1 | 🞎2 | 🞏3 | 🞎4 | 🞏5 |
| 51. Shift changes are problematic for patients in this hospital | 🞎1 | 🞎2 | 🞏3 | 🞎4 | 🞏5 |

**SECTION G: Number of Events Reported**

52. **In the past 12 months, how many event reports have you filled out and submitted?**

| 🞎 a. No event reports | 🞎 d. 6 to 10 event reports |
| --- | --- |
| 🞎 b. 1 to 2 event reports | 🞎 e. 11 to 20 event reports |
| 🞎 c. 3 to 5 event reports | 🞎 f. 21 event reports or more |

**SECTION H: Background Information**

**This information will help in the analysis of the survey results.**

53. Gender

| 🞎 a. Male | 🞎 b. Female |
| --- | --- |

54. Age

| 🞎 a. Less than 20 years | 🞎 d. 40 to 49 years |
| --- | --- |
| 🞎 b. 20 to 29 years | 🞎 e. 50 to 59 years |
| 🞎 c. 30 to 39 years | 🞎 f. 60 years or more |

55. How long have you worked in this hospital?

| 🞎 a. Less than 1 year | 🞎 d. 11 to 15 years |
| --- | --- |
| 🞎 b. 1 to 5 years | 🞎 e. 16 to 20 years |
| 🞎 c. 6 to 10 years | 🞎 f. 21 years or more |

56. How long have you worked in your current hospital work area/unit?

| 🞎 a. Less than 1 year | 🞎 d. 11 to 15 years |
| --- | --- |
| 🞎b. 1 to 5 years | 🞎 e. 16 to 20 years |
| 🞎 c. 6 to 10 years | 🞎 f. 21 years or more |

**SECTION H: Background Information (continued)**

57. Typically, how many hours per week do you work in this hospital?

| 🞎a. Less than 20 hours per week | 🞎d. 60 to 79 hours per week |
| --- | --- |
| 🞎 b. 20 to 39 hours per week | 🞎 e. 80 to 99 hours per week |
| 🞎c. 40 to 59 hours per week | 🞎 f. 100 hours per week or more |

58. In your staff position, do you typically have direct interaction or contact with patients?

| 🞎 a. YES, I typically have direct interaction or contact with patients. |
| --- |
| 🞎 b. NO, I typically do NOT have direct interaction or contact with patients. |

59. How long have you worked in your current specialty or profession?

| 🞎a. Less than 1 year | 🞎 d. 11 to 15 years |
| --- | --- |
| 🞎 b. 1 to 5 years | 🞎 e. 16 to 20 years |
| 🞎 c. 6 to 10 years | 🞎 f. 21 years or more |

**SECTION I: Your Comments**

**Please feel free to write any comments about patient safety, error, or event reporting in your hospital.**

|  |
| --- |

***THANK YOU FOR COMPLETING THIS SURVEY.***
